# Emotion dynamics as indicators of maladaptive emotions in patients with cancer

**DOI:** 10.1007/s00520-026-11320-7

**Published:** 2026-10-15

**Authors:** G. Elise Doppenberg-Smit, Femke Lamers, Annemarie M. J. Braamse, Hans Pruijt, Evelien J. M. Kuip, Inge M. Werter, Rune A. W. van de Wetering, Henk M. W. Verheul, Aartjan T. F. Beekman, Joost Dekker, Adriaan W. Hoogendoorn, Myra E. van Linde

**Affiliations:** 1 Department of Psychiatry, Vrije Universiteit Amsterdam, Amsterdam University Medical Center, Amsterdam, the Netherlands; 2 Mental Health Program, Amsterdam Public Health, Amsterdam, the Netherlands; 3 Cancer Treatment and Quality of Life, Cancer Center Amsterdam, Amsterdam, the Netherlands; 4 Department of Medical Psychology, Amsterdam University Medical Center, University of Amsterdam, Amsterdam, the Netherlands; 5 Department of Medical Oncology, Jeroen Bosch Hospital, Den Bosch, the Netherlands; 6 Department of Medical Oncology, Radboud Medical Center, Nijmegen, the Netherlands; 7 Pain and Palliative Care, Department of Anesthesiology, Radboud Medical Center, Nijmegen, the Netherlands; 8 Department of Medical Oncology, Rijnstate Hospital, Arnhem, the Netherlands; 9 Department of Medical Oncology, Slingeland Ziekenhuis, Doetinchem, the Netherlands; 10 Department of Medical Oncology, Erasmus Medical Center, Rotterdam, the Netherlands; 11 Department of Medical Oncology, Amsterdam University Medical Center, Vrije Universiteit Amsterdam, Amsterdam, the Netherlands

**Keywords:** Emotion dynamics, Cancer, Symptoms, Adaptation

## Abstract

**Purpose:**

Patients with cancer frequently experience a wide range of emotions in response to their diagnosis and treatment. Emotions are essentially adaptive, helping patients to cope with the disease. In a minority of patients, however, emotions may become maladaptive and warrant professional mental healthcare. In the field of psycho-oncology, the intensity of emotions is used to distinguish between adaptive and maladaptive emotions. Emotion dynamics (that is changes in emotions over time) may provide additional information on the adaptiveness of the emotions. The aim of this study was to investigate whether emotion dynamics (specifically inertia, variability, and instability) as well as emotion intensity are indicators of maladaptive emotions.

**Method:**

Patients with a solid malignancy (metastatic and non-metastatic disease; *n* = 132) were categorized as having adaptive or maladaptive emotions based on one of two reference standards: the psychiatric diagnostic assessment or the subjective need for professional mental healthcare. Patients reported on eight negative emotional symptoms for 14 days using Ecological Momentary Assessment on their mobile phone.

Autocorrelation (inertia), standard deviation (variability), root mean square successive differences (instability), and emotional intensity were compared between patients with adaptive or maladaptive emotions using *t*-tests.

**Results:**

Variability and instability as well as intensity were higher in patients with maladaptive emotions, compared to patients with adaptive emotions. Little evidence was found that patients with maladaptive emotions experienced more emotional inertia.

**Conclusion:**

This is the first study to report that in patients with cancer, emotional instability and variability are indicators of maladaptive emotions, in addition to the intensity of emotions.

**Supplementary Information:**

The online version contains supplementary material available at https://doi.org/10.1007/s00520-026-11320-7.

## Introduction

Patients with cancer frequently experience a wide range of emotions in response to their diagnosis and treatment. An emotion is a complex reaction pattern, involving experiential, behavioral, and physiological elements [1]. Emotions are adaptive in nature; they serve the purpose to cope with events [1]. For example, feelings of sadness or anxiety may help to deal with the risks associated with the diagnosis of cancer and to adhere to treatment. In a minority of patients, however, emotions may reflect a dysfunction in mental functioning, causing the patient harm and hindering daily functioning and the ability to cope effectively with the disease [2]. Examples include extreme anxiety, leading to the avoidance of scans and tests, or prolonged episodes of sadness despite positive treatment outcomes. These emotions are maladaptive and warrant professional mental healthcare [3]. Therefore, valid indicators for maladaptive emotions are needed to identify these patients.

Current measurement methods such as the Distress Thermometer assess the intensity of emotions. However, many patients who score above the validated cut-off indicating distress do not wish to receive a referral or do not wish to utilize psychological care [4–6]. Additional indicators may help to better distinguish between patients with adaptive emotions who do not need psychological care, and patients with maladaptive emotions who do need psychological care. Emotions are not static: the dynamics of emotions may provide additional information on the adaptiveness of emotions [7–9]. In the field of emotion dynamics, three indicators have been thoroughly examined and consistently linked to psychological well-being and psychopathology [7, 10]. These three indicators are emotional inertia, emotional variability, and emotional instability. *Emotional inertia* refers to emotions that are resistant to change [9, 11]. Emotions from a given moment carry over to the next moment, meaning they linger and perpetuate over time. Emotional inertia is most often calculated by autocorrelations [10]. *Emotional variability* refers to the range of emotions. A person with a high range will have emotions that are more extreme as compared to a person with a low range [10]. Emotional variability is most often calculated by standard deviation [8], and a person with high emotional variability will deviate more from their average emotions [10]. *Emotional instability* refers to the magnitude of the emotional changes. A person with a high emotional instability experiences rapid shifts in emotions, resulting in unstable emotions [10]. Emotional instability is often calculated by the root mean squared successive difference (RMSSD) [12].

Emotion dynamics are well-studied in patients with psychopathology, such as major depressive disorder [13], anxiety disorder [10], borderline personality disorder [7], or neuroticism [14], providing insight into how emotion dynamics differ between patients with and without psychopathology. However, to the best of our knowledge, emotion dynamics have not yet been studied in patients with cancer. Therefore, the current study aims to examine whether indicators of maladaptive emotions in patients with cancer can be derived from the dynamics of emotions. We hypothesize that patients with maladaptive emotions are characterized by emotional symptoms that (a) linger and perpetuate more across time (emotional inertia), (b) are more extreme (emotional variability), (c) are more unstable (emotional instability), or (d) more intense (emotional intensity), compared to patients with adaptive emotions.

## Methods

### Design

This observational study including patients with cancer took place at four academic and two nonacademic hospitals in the Netherlands between August 2021 and April 2024. Negative emotional symptoms were assessed with Ecological Momentary Assessment (EMA) that patients filled out for 2 weeks. The presence of adaptive versus maladaptive emotions was assessed with a reference standard: (a) psychiatric diagnostic assessment or (b) the patient’s subjective need for professional mental healthcare. Patients started their assessment 3–7 months after start of systemic treatment; all assessments were repeated after 3 months. The study was registered on clinicaltrials.gov (NCT05062447) and was approved by the Medical Ethics Committee of Amsterdam UMC (2020.099-NL72824.029.20). All participants provided written informed consent.

### Sample

Patients were eligible if they received a diagnosis of a malignant solid tumor (metastasized or non-metastasized), started with (neo)adjuvant or first-line chemo- or immunotherapy 3–7 months prior to participation, and had a > 3-month life expectancy. Exclusion criteria were as follows: age < 18; no sufficient understanding of the Dutch language; the presence of psychopathology other than mood or anxiety disorder (e.g., lifetime history of bipolar disorder, schizophrenia, major depressive disorder with psychotic features, schizoaffective disorders); severe cognitive impairments (e.g., dementia); (indication of) brain tumor or brain metastases; a history of severe drug or alcohol abuse within the past 6 months; not competent in the use of a mobile phone; and providing no informed consent. Patients were approached by their treating oncologist or nurse (specialist) for enrollment in this study.

### Screening

We aimed to include 80 patients with adaptive and 80 with maladaptive emotions (see power calculation). Because more patients experience adaptive emotions, after 80% of patients with adaptive emotions were included, we screened for patients with maladaptive emotions using the extended version of the Kessler-10 (EK-10) [15] and with the subjective need for professional mental healthcare question. Going forward, patients could only be included if they were screen-positive on the EK-10 (a score ≥ 20, or a positive answer on one of the five additional questions indicating distress [15]), or if they indicated to currently have or wished to have a referral because of their emotional symptoms.

### Measures

Sociodemographic information was collected using a self-reported online questionnaire developed for this study. Medical information including cancer diagnosis, metastasis (yes/no), timing of diagnosis, and first treatment were retrieved from the medical record.

### Reference standards

Patients were categorized as experiencing either adaptive or maladaptive emotions, using one of two reference standards. (a) *Psychiatric diagnostic assessment.* The Composite International Diagnostic Interview version 2.1 (CIDI) section Anxiety and Depression is a full-structured diagnostic interview with high interrater reliability to assess current (1-month recency) and lifetime presence of a depressive or anxiety disorder according to the DSM-IV criteria [16]. A trained researcher (ED) or research assistant conducted the interviews, and supervision of a psychiatrist (ATFB) was available. The Four Dimensional Symptom Questionnaire (4DSQ) was used to assess adjustment disorder [17]. Patients who scored > 20 on the Distress scale and did not have an anxiety or depression disorder according to the CIDI were categorized as having an adjustment disorder. Patients with a current anxiety or depressive disorder according to the CIDI or an adjustment disorder according to the 4DSQ were grouped as having maladaptive emotions. (b) *Subjective need for professional mental healthcare.* The Problem List was used to assess the subjective need for professional mental healthcare [18]. The Problem List is a self-reported questionnaire to assess problems in multiple domains [18]. The emotional domain was used to measure subjective need for professional mental healthcare. Patients indicated if they experienced a problem in this domain over the past 3 months, using a 5-point scale (ranging from 1 “no problem at all” to 5 “very much a problem”), as was done in previous studies [19, 20]. When participants reported at least one emotional problem, they were asked whether they (i) received a referral for the problem, (ii) did not receive a referral, but would have preferred a referral for the problem, (iii) did not want or did not receive a referral for the problem, but would be willing to consider a referral in the future, or (iv) did not want or received a referral, neither in the present nor the future. If patients had received a referral or would have preferred to have received a referral for emotional problems as indicated on the Problem List, they were grouped as having maladaptive emotions [19, 20].

### Emotional symptoms

Momentary emotional symptoms were documented via mobile phone during 14 consecutive days, 5 times per day using EMA. Patients received a text message with a personalized link to the EMA using the secured server system RoQua [21]. A fixed assessment protocol was used to get time-series data with equidistant time points [22]. Patients were instructed to respond to the prompt as quickly as possible and were given 60 minutes to respond [22]. A reminder was sent after 30 minutes. The full EMA protocol consisted of 15 emotional and 6 cancer-treatment-related symptoms, followed by 5 questions on activities since the last assessment, and where and with whom they were at the moment of the assessment. Only the negative emotional symptoms were used in the current analyses. Negative emotions were rated on a 7-point scale (1 = not at all; 7 = very much) and included feeling irritated, upset, listless/apathetic, down, nervous, bored, anxious, and worried. The emotions as well as the assessment protocol (14 consecutive days, 5 times per day) were derived from the Netherlands Study of Depression and Anxiety (NESDA) EMA diary-protocol [23] and have been widely used in previous studies [24–27]*.*

### Statistical analyses

All analyses were conducted in R (version 4.4.3) [28]. All three emotion parameters were operationalized in the common way [10]. For each of the eight emotional symptoms, the autocorrelation (AC) across the 70 EMA was determined to indicate emotional inertia. The standard deviation (SD) was determined to indicate emotional variability. The root mean square successive difference (RMSSD) was determined to indicate emotional instability across the 70 EMA. Missings were not imputed, ACs and RMSSDs were computed from available pairs. The AC, SD, and RMSSD for each of the eight emotional symptoms were compared between patients with adaptive and maladaptive emotions using a Welch *t*-test, which can deal with unequal variance between samples. All *t*-tests were done twice, once with the grouping according to the psychiatric diagnostic assessment and once with the grouping according to the professional mental healthcare reference standard. Cohen’s *d* was calculated as effect size. Cohen’s *d* = 0.20 was considered a small effect, *d* = 0.50 was considered a moderate effect, and *d* = 0.80 was considered a large effect [29]. The analyses were conducted on the first assessment (3–7 months after start of treatment). Additional analyses were performed on patients with metastatic disease and non-metastatic disease separately and on the sample of patients who participated in the second measurement (3 months after the first assessment).

### Power calculation

A meta-analysis (with data on *N* = 11.381 subjects) has yielded estimates on the expected association of psychological well-being/psychopathology with emotional inertia, emotional variability, and emotional instability [10]. Using the data on emotional symptoms with a negative valence, the meta-analysis suggests an effect size correlation of 0.28, on average. This corresponds to Cohen’s *d* = 0.49 (that is, a medium effect size). For each patient, scores on emotional symptoms are available five times a day, during 14 days. With 14 observations per patient, assuming the intra-subject correlation equals 0.40, setting alpha = 0.05, and the power (1–beta) = 0.90, the total sample size requirement equals 78 [30]. Therefore, we aimed to recruit 40 patients with adaptive emotions and 40 patients with maladaptive emotions that is as follows: 80 patients in each stratum of non-metastatic disease or metastatic disease, therefore 160 patients in total.

## Results

Of the 134 included patients, 132 patients had sufficient EMA (< 50% missing) to be included in the analyses. The ratio of patients with maladaptive versus adaptive emotions was 1 to 4 for both reference standards. For patients’ characteristics, see Table 1. The response rate of the EMA was 93% across the targeted measurements: of the 9240 measurements, 8603 were filled out.

**Table 1:** Patient characteristics

	*n* (%)/*mean (SD)*
Age (range 22–86)	*57.7 (12.9)*
Gender	
Female	77 (58.3%)
Male	55 (41.7%)
Diagnosis	
Gastro-intestinal cancer	34 (25.8%)
Breast cancer	33 (25.0%)
Melanoma	31 (23.5%)
Urogenital cancer	12 (9.1%)
Gynecological cancer	8 (6.1%)
Sarcoma	7 (5.3%)
Head/neck cancer	5 (3.8%)
Other	2 (1.5%)
Metastatic disease	
Yes	58 (43.9%)
No	74 (56.1%)
Psychological help in past year	
No	100 (75.8%)
Yes	32 (24.2%)
Psychiatric diagnostic interview	
Adaptive emotions	101 (76.5%)
Maladaptive emotions	31 (23.5%)
Subjective need for help	
Adaptive emotions	97 (73.5%)
Maladaptive emotions	35 (26.5%)

*N* = 132. Information from this table has already been published in [31, 32]. Despite the same total number of participants, there are slight differences between the tables due to study-specific selection criteria

### Emotional intensity

The average score on emotional symptoms ranged from 1.28 to 3.04. All emotional symptoms were rated to be more intense by patients with maladaptive emotions than patients with adaptive emotions according to the psychiatric diagnostic assessment as reference standard (Cohen’s *d* = 0.58–1.06). For the subjective need for professional mental healthcare as reference standard, the differences were less pronounced. Whereas all symptoms appeared to be rated more intense by patients with maladaptive emotions as compared with patients with adaptive emotions (Cohen’s *d* = 0.22–0.65), only half were statistically significant (see Table 2).

**Table 2:** Intensity of emotional symptoms in patients with adaptive and maladaptive emotions

Emotional symptom	Group	Mean	SD	*t*-test, *p*-value	Cohen’s *d*	Emotional symptom	Group	Mean	SD	*t*-test, *p*-value	Cohen’s *d*
Psychiatric diagnostic assessment reference standard						Subjective need for professional mental healthcare reference standard					
Anxious	Adaptive emotions	1.33	0.63	*t* (39.16) = 3.00, *p* = 0.005	0.67	Anxious	Adaptive emotions	1.33	0.60	*t* (44.14) = 2.62, *p* = 0.01	0.57
	Maladaptive emotions	1.85	0.91				Maladaptive emotions	1.78	0.96		
	Total	1.45	0.74				Total	1.45	0.74		
Bored	Adaptive emotions	1.29	0.43	*t* (33.99) = 3.14, *p* = 0.004	0.75	Bored	Adaptive emotions	1.38	0.60	*t* (54.56) = 1.28, *p* = 0.207	0.26
	Maladaptive emotions	1.83	0.93				Maladaptive emotions	1.54	0.68		
	Total	1.42	0.62				Total	1.42	0.62		
Down	Adaptive emotions	1.54	0.70	*t* (37.03) = 4.61, *p* < 0.001	1.06	Down	Adaptive emotions	1.61	0.79	*t* (46.59) = 3.04, *p* = 0.004	0.65
	Maladaptive emotions	2.54	1.15				Maladaptive emotions	2.23	1.12		
	Total	1.77	0.93				Total	1.77	0.93		
Irritated	Adaptive emotions	1.37	0.54	*t* (37.96) = 2.76, *p* = 0.009	0.63	Irritated	Adaptive emotions	1.39	0.55	*t* (45.19) = 1.98, *p* = 0.053	0.43
	Maladaptive emotions	1.81	0.84				Maladaptive emotions	1.69	0.83		
	Total	1.47	0.65				Total	1.47	0.65		
Listless	Adaptive emotions	1.99	0.97	*t* (42.26) = 2.68, *p* = 0.01	0.58	Listless	Adaptive emotions	2.08	1.04	*t* (55.52) = 1.07, *p* = 0.291	0.22
	Maladaptive emotions	2.63	1.23				Maladaptive emotions	2.31	1.15		
	Total	2.14	1.07				Total	2.14	1.07		
Nervous	Adaptive emotions	1.43	0.65	*t* (39.01) = 3.15, *p* = 0.003		Nervous	Adaptive emotions	1.47	0.67	*t* (47.51) = 2.12, *p* = 0.039	0.45
	Maladaptive emotions	2.00	0.94		0.71		Maladaptive emotions	1.83	0.93		
	Total	1.57	0.76				Total	1.57	0.76		
Upset	Adaptive emotions	1.25	0.44	*t* (36.06) = 2.90, *p* = 0.006	0.67	Upset	Adaptive emotions	1.28	0.49	*t* (46.42) = 2.06, *p* = 0.045	0.44
	Maladaptive emotions	1.68	0.78				Maladaptive emotions	1.55	0.71		
	Total	1.35	0.57				Total	1.35	0.57		
Worried	Adaptive emotions	1.99	1.07	*t* (41.46) = 3.90, *p* < 0.001	0.85	Worried	Adaptive emotions	2.12	1.22	*t* (60.39) = 1.74, *p* = 0.088	0.34
	Maladaptive emotions	3.04	1.39				Maladaptive emotions	2.54	1.22		
	Total	2.24	1.23				Total	2.24	1.23		

*N* (adaptive) = 101, *N* (maladaptive) = 31, *N* (total) = 132 for the psychiatric diagnostic assessment as reference standard; *N* (adaptive) = 97, *N* (maladaptive) = 35, *N* (total) = 132 for the subjective need for professional mental healthcare as reference standard

### Emotional inertia

**Fig. 1:**
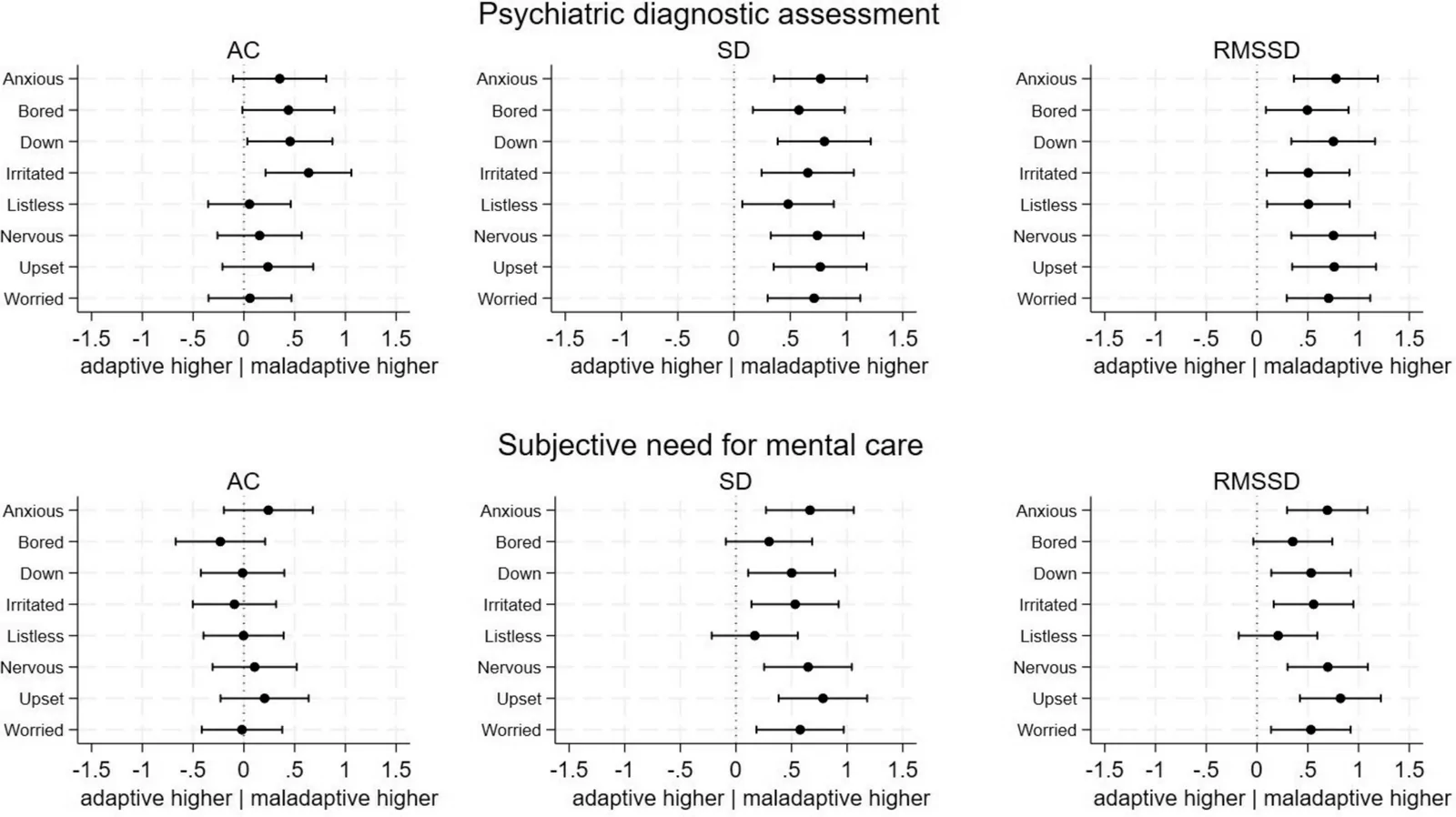
Cohen’s *d* effect sizes with 95% confidence intervals for group differences in autocorrelation (AC), standard deviation (SD), and root mean square successive difference (RMSSD) across eight emotional symptoms for the psychiatric diagnostic assessment and for the subjective need for mental healthcare reference standard. *Psychiatric diagnostic assessment *= psychiatric diagnostic assessment as reference standard; *subjective need for mental care* = subjective need for professional mental healthcare as reference standard

In 17.9% of the person/symptom combinations, the standard deviation was zero across all 70 EMA (see below). Because the autocorrelation is undetermined when a patient has no standard deviation across all EMA, only patients’ measurements with positive standard deviation were included in the analysis. Emotional inertia was higher for Irritated (Cohen’s *d* = 0.62) and Down (Cohen’s *d* = 0.45) for patients with maladaptive emotions as compared to adaptive emotions with the psychiatric diagnostic assessment as reference standard (see Fig. 1 and Supplemental Table 1). For the other six symptoms, no significant difference was found. With the subjective need for professional mental healthcare as reference standard, no statistically significant differences in emotional inertia were found between patients with adaptive and maladaptive emotions.

The analyses revealed that across the eight emotional symptoms and 132 patients (8 × 132 = 1056 instances), 189 (17.9%) instances occurred where the standard deviation was zero across the 70 EMA. Further examination showed that in 188 of the 189 instances, patients filled in a “1” (= not at all) for all 70 EMA for a given symptom, indicating that the symptom was not present at all during the two-week assessment period. These low stable levels of emotions were present in patients with adaptive as well as patients with maladaptive emotions (see Supplemental Table 2). For the psychiatric diagnostic assessment reference standard, the proportion of patients with low stable levels of emotions was significantly higher in the group of patients with adaptive emotions (8–33%) as compared to maladaptive emotions (0–13%) for all emotional symptoms, except for Anxious and Bored. For the subjective need for professional healthcare standard, the proportion of patients with low stable levels of emotions did not differ between patients with adaptive or maladaptive emotions, except for Upset, where more patients with low stable levels of emotions were present in the group with adaptive emotions (33%) than in the group with maladaptive emotions (14%).

### Emotional variability

Emotional variability (SD) was higher for all eight emotional symptoms for patients with maladaptive emotions as compared to adaptive emotions with the psychiatric diagnostic assessment as reference standard (see Fig. 1 and Supplemental Table 3). Cohen’s *d* ranged from 0.49 to 0.84, indicating moderate to large effects. With the subjective need for professional mental healthcare as reference standard, patients with maladaptive emotions had significantly higher emotional variability across the 70 EMA for all symptoms apart from Bored and Listless. Cohen’s *d* for the six symptoms that were higher for patients with maladaptive emotions ranged from 0.50 to 0.75, indicating moderate effects.

### Emotional instability

The results for instability (RMSSD) were analogous to the results for variability. Emotional instability was higher for all eight emotional symptoms for patients with maladaptive emotions as compared to adaptive emotions with the psychiatric diagnostic assessment as reference standard (see Fig. 1 and Supplemental Table 4). Cohen’s *d* ranged from 0.48 to 0.79, indicating moderate to large effects. With the subjective need for professional mental healthcare as reference standard, patients with maladaptive emotions had significantly higher emotional instability across the 70 EMA for all symptoms apart from Bored and Listless. Cohen’s *d* for the six symptoms that were higher for patients with maladaptive emotions ranged from 0.55–0.80, indicating moderate to large effects.

### Metastatic versus non-metastatic disease

The analyses were repeated separately in patients with metastatic disease and those without metastatic disease. The results were similar to those of the total sample. The confidence intervals were larger in these analyses because of the reduced sample size, sometimes resulting in non-significant differences (see Supplemental Figs. 1 and 2).

### Second assessment

The analyses were repeated in the subset of patients who participated in the second measurement (*n* = 110 for the psychiatric diagnostic assessment as reference standard; *n* = 105 for the subjective need for professional mental healthcare as reference standard). The results were similar to those of the first assessment where the SD and RMSSD were higher in patients with maladaptive emotions and with AC showing both significant and non-significant differences (see Supplemental Fig. 3).

## Discussion

This study examined the differences in emotion dynamics in patients with cancer with adaptive and maladaptive emotions using EMA data on emotional symptoms. The key findings were (a) as hypothesized, patients with maladaptive emotions showed higher instability and higher variability in emotional symptoms; (b) except for two symptoms, no differences were found in emotional inertia between patients with adaptive and maladaptive emotions, which contrasted with our hypothesis; and (c) patients with maladaptive emotions experienced more intense emotional symptoms as compared to patients with adaptive emotions; this was the case for all symptoms according to the psychiatric diagnostic assessment as reference standard, and for half of the symptoms for the subjective need for professional mental healthcare as reference standard.

Patients with maladaptive emotions showed emotional symptoms that were higher in instability and variability compared to patients with adaptive emotions. This is in line with studies in patients with psychopathology who were also characterized by more extreme and unstable emotions [13, 14, 23, 33]. To our knowledge, this is the first study to report that these associations apply to patients with cancer.

Patients with adaptive and maladaptive emotions only differed in emotional inertia in two symptoms according to the psychiatric diagnostic assessment as reference standard and no differences were found according to the subjective need for professional mental healthcare as reference standard. This may partly be because a proportion of patients showed low stable levels of emotional symptoms. However, previous studies on patients with psychopathology showed mixed results regarding inertia. The association between inertia and for example depression has been found in certain studies [10, 23, 33, 34] but not in others [13, 35, 36]. Therefore, the status of inertia as an indicator of maladaptive emotions is uncertain.

Finally, patients with maladaptive emotions experience more intense emotional symptoms than patients with adaptive emotions. Intensity was measured by the average of emotional symptoms across 70 moments in a two-week timespan. Our results confirm the results of studies in which intensity was measured at a single fixed moment in time [18]. Remarkably, the differences between patients with adaptive and maladaptive emotions were larger in the analysis using the psychiatric diagnostic assessment as reference standard than the analysis using the subjective need for professional mental healthcare as reference standard. The differences were both larger in effect size and in the number of symptoms that showed significant differences (all versus half of the symptoms). These results suggest that the intensity of emotions is especially an indicator of maladaptive emotions in patients with anxiety or depressive disorder and less so for patients with a subjective need for professional mental healthcare. This finding asks for replication in further research.

Strengths of this study firstly include the use of EMA because it captures momentary emotions over a longer time period. Secondly, this study used two reference standards to group patients as having adaptive or maladaptive emotions. Apparently, the results are robust and not dependent on a specific reference standard. This study also has limitations that need to be considered. First, because there were more patients with low stable levels of symptoms than anticipated, the sample size to analyze emotional inertia was therefore lower than expected. This may have reduced the power of this part of the study, asking for further replication. Second, the group of patients with maladaptive emotions was smaller than the group with adaptive emotions, even after screening for patients with maladaptive emotions. Likewise, this may have reduced the power. However, despite potentially lower statistical power a priori, statistically significant effects were consistently found for emotional instability and variability. Nevertheless, Fig. 1 shows wide confidence intervals, leading to considerable uncertainty about the effects. This further emphasizes the need for replication. Third, we have used the common operationalization of emotion dynamics parameters [10]. However, other operationalizations are possible.

The clinical implications are that in addition to the intensity of emotions, instability and variability are also indicators of maladaptive emotions. Further research using new samples is needed to determine the best way to do this. For instability, variability, as well as intensity, the threshold value and diagnostic accuracy need to be determined. In addition to examining each indicator individually, cutoff value and diagnostic accuracy can also be analyzed for a combination of these indicators. Subsequently, the integration of these indicators into broader symptom monitoring methods needs to be investigated. In this regard, it is particularly important to determine how useful these indicators are to clinicians in distinguishing between patients with adaptive and maladaptive emotions, in addition to their own clinical impression of the patient. Further training in the use of these indicators may be valuable in this regard. The added value of these indicators in clinical practice needs to be determined.

In conclusion, this is the first study to report that emotional instability and variability are indicators of maladaptive emotions in patients with cancer, in addition to the intensity of emotions. Little support was found for the hypothesis that patients with maladaptive emotions experience higher emotional inertia.

## Supplementary Information

Below is the link to the electronic supplementary material.

S Fig. 1 (DOCX 4.12 MB)

S Fig. 2 (DOCX 4.12 MB)

S Fig. 3 (DOCX 4.12 MB)

S Table 1 (DOCX 32.5 KB)

S Table 2 (DOCX 34.0 KB)

S Table 3 (DOCX 34.1 KB)

S Table 4 (DOCX 34.0 KB)

## Data Availability

The data are available upon reasonable request.

## Code availability

Available upon reasonable request
