# Supplementary material for: Emotion dynamics as indicators of maladaptive emotions in patients with cancer
Source: Support Care Cancer. 2026 Oct 15;34(11):1103. doi: 10.1007/s00520-026-11320-7 (PMC13672517; doi:10.1007/s00520-026-11320-7)
Supplement: Supplementary file 1 [file 520_2026_11320_MOESM1_ESM.docx]

**Supplemental Fig. 1** Cohen’s d effect sizes with 95% confidence intervals for group differences in autocorrelation (AC), standard deviation (SD), and root mean square successive difference (RMSSD) across eight emotional symptoms for the psychiatric diagnostic assessment and for the subjective need for mental healthcare reference standard for patients with non-metastatic disease. Psychiatric diagnostic assessment = psychiatric diagnostic assessment reference standard; Subjective need for mental care = subjective need for professional mental healthcare reference standard
