# Supplementary material for: Emotion dynamics as indicators of maladaptive emotions in patients with cancer
Source: Support Care Cancer. 2026 Oct 15;34(11):1103. doi: 10.1007/s00520-026-11320-7 (PMC13672517; doi:10.1007/s00520-026-11320-7)
Supplement: Supplementary file 4 [file 520_2026_11320_MOESM4_ESM.docx]

Supplemental Table 1. Autocorrelation of emotional symptoms in patients with adaptive and maladaptive emotions.

| Emotional  Symptom | Group | n | Mean | SD | t- test, p-value | Cohen’s d | Emotional  Symptom | Group | n | Mean | SD | t- test, p-value | Cohen’s d |
| --- | --- | --- | --- | --- | --- | --- | --- | --- | --- | --- | --- | --- | --- |
| Psychiatric diagnostic assessment reference standard | | | | | | | Subjective need for professional mental healthcare reference standard | | | | | | |
| Anxious | Adaptive emotions | 70 | 0.19 | 0.24 | t(45.59) = 1.58,  p = 0.122 | 0.36 | Anxious | Adaptive emotions | 66 | 0.19 | 0.25 | t(62.34)= 1.15,  p = 0.253 | 0.25 |
|  | Maladaptive emotions | 25 | 0.27 | 0.23 |  |  |  | Maladaptive emotions | 29 | 0.25 | 0.21 |  |  |
| Bored | Adaptive emotions | 71 | 0.12 | 0.20 | t(38.95) = 1.78,  p = 0.083 | 0.42 | Bored | Adaptive emotions | 69 | 0.16 | 0.23 | t(63.19) = -1.14,  p = 0.258 | -0.24 |
|  | Maladaptive emotions | 26 | 0.21 | 0.24 |  |  |  | Maladaptive emotions | 28 | 0.11 | 0.18 |  |  |
| Down | Adaptive emotions | 80 | 0.23 | 0.23 | t(53.69) = 2.13,  p = 0.038 | 0.45 | Down | Adaptive emotions | 79 | 0.26 | 0.24 | t(60.48) = -0.06,  p = 0.952 | -0.01 |
|  | Maladaptive emotions | 31 | 0.33 | 0.23 |  |  |  | Maladaptive emotions | 32 | 0.26 | 0.22 |  |  |
| Irritated | Adaptive emotions | 81 | 0.11 | 0.20 | t(48.46) = 2.84,  p = 0.007 | 0.62 | Irritated | Adaptive emotions | 80 | 0.15 | 0.22 | t(63.76) = -0.47,  p = 0.642 | --0.10 |
|  | Maladaptive emotions | 31 | 0.24 | 0.23 |  |  |  | Maladaptive emotions | 32 | 0.13 | 20 |  |  |
| Listless | Adaptive emotions | 93 | 0.21 | 0.21 | t(49.02) = 0.26,  p = 0.797 | 0.05 | Listless | Adaptive emotions | 90 | 0.21 | 0.21 | t(55.39) = -0.02,  p = 0.988 | -0.00 |
|  | Maladaptive emotions | 31 | 0.22 | 0.22 |  |  |  | Maladaptive emotions | 34 | 0.21 | 0.23 |  |  |
| Nervous | Adaptive emotions | 81 | 0.19 | 0.21 | t(55.14) = 0.74,  p = 0.464 | 0.16 | Nervous | Adaptive emotions | 81 | 0.20 | 0.22 | t(62.89) = 0.54,  p = 0.594 | 0.11 |
|  | Maladaptive emotions | 31 | 0.23 | 0.21 |  |  |  | Maladaptive emotions | 31 | 0.22 | 0.19 |  |  |
| Upset | Adaptive emotions | 68 | 0.18 | 0.22 | t(52.05) = 1.08,  p = 0.283 | 0.24 | Upset | Adaptive emotions | 65 | 0.18 | 0.21 | t(55.11) = 0.92,  p = 0.363 | 0.20 |
|  | Maladaptive emotions | 27 | 0.23 | 0.21 |  |  |  | Maladaptive emotions | 30 | 0.23 | 0.22 |  |  |
| Worried | Adaptive emotions | 90 | 0.25 | 0.26 | t(57.91) = 0.31,  p = 0.761 | 0.06 | Worried | Adaptive emotions | 87 | 0.26 | 0.26 | t(66.09) = -0.09, p = 0.924 | -0.02 |
|  | Maladaptive emotions | 31 | 0.27 | 0.23 |  |  |  | Maladaptive emotions | 34 | 0.25 | 0.23 |  |  |
