# Supplementary material for: Emotion dynamics as indicators of maladaptive emotions in patients with cancer
Source: Support Care Cancer. 2026 Oct 15;34(11):1103. doi: 10.1007/s00520-026-11320-7 (PMC13672517; doi:10.1007/s00520-026-11320-7)
Supplement: Supplementary file 5 [file 520_2026_11320_MOESM5_ESM.docx]

Supplemental Table 2. Proportion of patients with low stable levels of emotions in patients with adaptive and maladaptive emotions

| Emotional  Symptom | Group | n | Mean | SD | t- test, p-value | Cohen’s d | Emotional  Symptom | Group | n | Mean | SD | t- test, p-value | Cohen’s d |
| --- | --- | --- | --- | --- | --- | --- | --- | --- | --- | --- | --- | --- | --- |
| Psychiatric diagnostic assessment reference standard | | | | | | | Subjective need for professional mental healthcare reference standard | | | | | | |
| Anxious | Adaptive emotions | 101 | 0.31 | 0.46 | t(56.70) = -1.32, p = 0.191 | -0.26 | Anxious | Adaptive emotions | 97 | 0.32 | 0.47 | t(73.24) = -1.85,  p = 0.069 | -0.35 |
|  | Maladaptive emotions | 31 | 0.19 | 0.40 |  |  |  | Maladaptive emotions | 35 | 0.17 | 0.38 |  |  |
| Bored | Adaptive emotions | 101 | 0.30 | 0.46 | t(60.34) = -1.67, p = 1.67 | -0.32 | Bored | Adaptive emotions | 97 | 0.29 | 0.46 | t(67.03) = -1.07,  p = 0.288 | -0.21 |
|  | Maladaptive emotions | 31 | 0.16 | 0.37 |  |  |  | Maladaptive emotions | 35 | 0.20 | 0.41 |  |  |
| Down | Adaptive emotions | 101 | 0.21 | 0.41 | t(100) = -5.12, p<0.001 | -0.72 | Down | Adaptive emotions | 97 | 0.19 | 0.39 | t(82.65) = -1.60,  p = 0.113 | -0.29 |
|  | Maladaptive emotions | 31 | 0.00 | 0.00 |  |  |  | Maladaptive emotions | 35 | 0.09 | 0.28 |  |  |
| Irritated | Adaptive emotions | 101 | 0.20 | 0.40 | t(100) = -4.97, p<0.001 | -0.70 | Irritated | Adaptive emotions | 97 | 0.18 | 0.38 | t(80.73) = -1.45,  p = 0.151 | -0.27 |
|  | Maladaptive emotions | 31 | 0.00 | 0.00 |  |  |  | Maladaptive emotions | 35 | 0.09 | 0.28 |  |  |
| Listless | Adaptive emotions | 101 | 0.08 | 0.27 | t(100) = -2.93,  p = 0.004 | -0.41 | Listless | Adaptive emotions | 97 | 0.07 | 0.26 | t(92.90) = -1.12,  p = 0.265 | -0.20 |
|  | Maladaptive emotions | 31 | 0.00 | 0.00 |  |  |  | Maladaptive emotions | 35 | 0.03 | 0.17 |  |  |
| Nervous | Adaptive emotions | 101 | 0.20 | 0.40 | t(100) = -4.97, p<0.001 | -0.70 | Nervous | Adaptive emotions | 97 | 0.16 | 0.37 | t(68.99) = -0.76,  p = 0.448 | -0.15 |
|  | Maladaptive emotions | 31 | 0.00 | 0.00 |  |  |  | Maladaptive emotions | 35 | 0.11 | 0.32 |  |  |
| Upset | Adaptive emotions | 101 | 0.33 | 0.47 | t(68.49) = -2.56,  p = 0.013 | -0.48 | Upset | Adaptive emotions | 97 | 0.33 | 0.47 | t(79.82) = -2.43,  p = 0.017 | -0.45 |
|  | Maladaptive emotions | 31 | 0.13 | 0.34 |  |  |  | Maladaptive emotions | 35 | 0.14 | 0.36 |  |  |
| Worried | Adaptive emotions | 101 | 0.11 | 0.31 | t(100) = -3.50,  p<0.001 | -0.49 | Worried | Adaptive emotions | 97 | 0.10 | 0.31 | t(108.21) = -1.77,  p = 0.080 | -0.30 |
|  | Maladaptive emotions | 31 | 0.00 | 0.00 |  |  |  | Maladaptive emotions | 35 | 0.03 | 0.17 |  |  |
