# Supplementary material for: Emotion dynamics as indicators of maladaptive emotions in patients with cancer
Source: Support Care Cancer. 2026 Oct 15;34(11):1103. doi: 10.1007/s00520-026-11320-7 (PMC13672517; doi:10.1007/s00520-026-11320-7)
Supplement: Supplementary file 6 [file 520_2026_11320_MOESM6_ESM.docx]

Supplemental Table 3. Standard deviation of emotional symptoms in patients with adaptive and maladaptive emotions

| Emotional  Symptom | Group | n | Mean | SD | t- test, p-value | Cohen’s d | Emotional  Symptom | Group | n | Mean | SD | t- test, p-value | Cohen’s d |
| --- | --- | --- | --- | --- | --- | --- | --- | --- | --- | --- | --- | --- | --- |
| Psychiatric diagnostic assessment reference standard | | | | | | | Subjective need for professional mental healthcare reference standard | | | | | | |
| Anxious | Adaptive emotions | 101 | 0.40 | 0.42 | t(42.33) = 3.33,  p = 0.002 | 0.72 | Anxious | Adaptive emotions | 97 | 0.40 | 0.43 | t(52.59) = 3.12,  p = 0.003 | 0.64 |
|  | Maladaptive emotions | 31 | 0.74 | 0.52 |  |  |  | Maladaptive emotions | 35 | 0.70 | 0.50 |  |  |
| Bored | Adaptive emotions | 101 | 0.46 | 0.41 | t(46.64) = 2.69,  p = 0.010 | 0.56 | Bored | Adaptive emotions | 97 | 0.48 | 0.43 | t(58.79) = 1.49, p = 0.141 | 0.30 |
|  | Maladaptive emotions | 31 | 0.70 | 0.45 |  |  |  | Maladaptive emotions | 35 | 0.61 | 0.44 |  |  |
| Down | Adaptive emotions | 101 | 0.61 | 0.47 | t(57.49) = 4.26, p<0.001 | 0.84 | Down | Adaptive emotions | 97 | 0.63 | 0.47 | t(60.51) = 2.55,  p = 0.013 | 0.50 |
|  | Maladaptive emotions | 31 | 0.98 | 0.40 |  |  |  | Maladaptive emotions | 35 | 0.87 | 0.47 |  |  |
| Irritated | Adaptive emotions | 101 | 0.55 | 0.46 | t(48.46) = 3.14,  p = 0.003 | 0.65 | Irritated | Adaptive emotions | 97 | 0.56 | 0.45 | t(52.74) = 2.50,  p = 0.015 | 0.51 |
|  | Maladaptive emotions | 31 | 0.86 | 0.48 |  |  |  | Maladaptive emotions | 35 | 0.81 | 0.53 |  |  |
| Listless | Adaptive emotions | 101 | 0.83 | 0.46 | t(53.92) = 2.46,  p = 0.017 | 0.49 | Listless | Adaptive emotions | 97 | 0.86 | 0.48 | t(66.48) = 0.91,  p = 0.369 | 0.17 |
|  | Maladaptive emotions | 31 | 1.05 | 0.42 |  |  |  | Maladaptive emotions | 35 | 0.94 | 0.43 |  |  |
| Nervous | Adaptive emotions | 101 | 0.58 | 0.46 | t(53.35) = 3.76, p<0.001 | 0.76 | Nervous | Adaptive emotions | 97 | 0.58 | 0.44 | t(54.18) = 3.10,  p = 0.003 | 0.63 |
|  | Maladaptive emotions | 31 | 0.91 | 0.42 |  |  |  | Maladaptive emotions | 35 | 0.87 | 0.50 |  |  |
| Upset | Adaptive emotions | 101 | 0.40 | 0.39 | t(42.12) = 3.30,  p = 0.002 | 0.72 | Upset | Adaptive emotions | 97 | 0.39 | 0.39 | t(52.72) = 3.68,  p = 0.001 | 0.75 |
|  | Maladaptive emotions | 31 | 0.72 | 0.49 |  |  |  | Maladaptive emotions | 35 | 0.71 | 0.46 |  |  |
| Worried | Adaptive emotions | 101 | 0.60 | 0.43 | t(67.12) = 4.07, p<0.001 | 0.77 | Worried | Adaptive emotions | 97 | 0.60 | 0.42 | t(63.73) = 3.02,  p = 0.004 | 0.59 |
|  | Maladaptive emotions | 31 | 0.88 | 0.32 |  |  |  | Maladaptive emotions | 35 | 0.84 | 0.39 |  |  |
