# Supplementary material for: Emotion dynamics as indicators of maladaptive emotions in patients with cancer
Source: Support Care Cancer. 2026 Oct 15;34(11):1103. doi: 10.1007/s00520-026-11320-7 (PMC13672517; doi:10.1007/s00520-026-11320-7)
Supplement: Supplementary file 7 [file 520_2026_11320_MOESM7_ESM.docx]

Supplemental Table 4. Root mean square successive difference of emotional symptoms in patients with adaptive and maladaptive emotions

| Emotional  Symptom | Group | n | Mean | SD | t- test, p-value | Cohen’s d | | Emotional  Symptom | | Group | | n | | Mean | | SD | | t- test, p-value | | Cohen’s d | |
| --- | --- | --- | --- | --- | --- | --- | --- | --- | --- | --- | --- | --- | --- | --- | --- | --- | --- | --- | --- | --- | --- |
| Psychiatric diagnostic assessment reference standard | | | | | | | Subjective need for professional mental healthcare reference standard | | | | | | | | | | | | | |  |
| Anxious | Adaptive emotions | 101 | 0.44 | 0.44 | t(43.93) = 3.47,  p = 0.001 | 0.74 | Anxious | | Adaptive emotions | | 97 | | 0.44 | | 0.44 | | t(52.55) = 3.25,  p = 0.002 | | 0.67 | |  |
|  | Maladaptive emotions | 31 | 0.80 | 0.52 |  |  |  | | Maladaptive emotions | | 35 | | 0.76 | | 0.52 | |  |  |  | |  |
| Bored | Adaptive emotions | 101 | 0.56 | 0.50 | t(46.52) = 2.30,  p = 0.026 | 0.48 | Bored | | Adaptive emotions | | 97 | | 0.58 | | 0.50 | | t(56.75) = 1.73,  p = 0.090 | | 0.35 | |  |
|  | Maladaptive emotions | 31 | 0.82 | 0.54 |  |  |  | | Maladaptive emotions | | 35 | | 0.76 | | 0.54 | |  |  |  | |  |
| Down | Adaptive emotions | 101 | 0.67 | 0.49 | t(58.22) = 4.01,  p <0.001 | 0.79 | Down | | Adaptive emotions | | 97 | | 0.68 | | 0.49 | | t(61.95) = 2.75,  p = 0.008 | | 0.54 | |  |
|  | Maladaptive emotions | 31 | 1.03 | 0.42 |  |  |  | | Maladaptive emotions | | 35 | | 0.95 | | 0.48 | |  |  |  | |  |
| Irritated | Adaptive emotions | 101 | 0.68 | 0.57 | t(55.39) = 2.62,  p = 0.011 | 0.52 | Irritated | | Adaptive emotions | | 97 | | 0.66 | | 0.53 | | t(53.04) = 2.63,  p = 0.011 | | 0.54 | |  |
|  | Maladaptive emotions | 31 | 0.96 | 0.51 |  |  |  | | Maladaptive emotions | | 35 | | 0.97 | | 0.62 | |  |  |  | |  |
| Listless | Adaptive emotions | 101 | 0.95 | 0.51 | t(52.65) = 2.56,  p = 0.014 | 0.52 | Listless | | Adaptive emotions | | 97 | | 0.98 | | 0.52 | | t(66.20) = 1.11,  p = 0.272 | | 0.21 | |  |
|  | Maladaptive emotions | 31 | 1.20 | 0.48 |  |  |  | | Maladaptive emotions | | 35 | | 1.08 | | 0.47 | |  |  |  | |  |
| Nervous | Adaptive emotions | 101 | 0.66 | 0.51 | t(57.71) = 3.99,  p <0.001 | 0.78 | Nervous | | Adaptive emotions | | 97 | | 0.65 | | 0.48 | | t(54.30) = 3.34,  p = 0.002 | | 0.68 | |  |
|  | Maladaptive emotions | 31 | 1.03 | 0.43 |  |  |  | | Maladaptive emotions | | 35 | | 1.00 | | 0.54 | |  |  |  | |  |
| Upset | Adaptive emotions | 101 | 0.46 | 0.43 | t(43.49) = 3.36,  p = 0.002 | 0.72 | Upset | | Adaptive emotions | | 97 | | 0.44 | | 0.43 | | t(53.88) = 3.92,  p <0.001 | | 0.80 | |  |
|  | Maladaptive emotions | 31 | 0.80 | 0.51 |  |  |  | | Maladaptive emotions | | 35 | | 0.81 | | 0.49 | |  |  |  | |  |
| Worried | Adaptive emotions | 101 | 0.65 | 0.46 | t(81.57) = 4.38, p<0.001 | 0.79 | Worried | | Adaptive emotions | | 97 | | 0.66 | | 0.47 | | t(67.61) = 2.86,  p = 0.005 | | 0.55 | |  |
|  | Maladaptive emotions | 31 | 0.96 | 0.30 |  |  |  | | Maladaptive emotions | | 35 | | 0.90 | | 0.41 | |  |  |  | |  |
